# miR-548ag promotes DPP4 expression in hepatocytes through activation of TLR(7/8)/NF-κB pathway

**DOI:** 10.1038/s41366-024-01504-8

**Published:** 2024-02-29

**Authors:** Jianyu Xiong, Chaoyue Sun, Xin Wen, Yanting Hou, Maodi Liang, Jie Liu, Qianqian Wei, Fangyuan Yuan, Chaoling Peng, Yao Chen, Yongsheng Chang, Cuizhe Wang, Jun Zhang

**Affiliations:** 1 Medical College of Shihezi University, Bei-Er-Lu, Shihezi, 832000 Xinjiang China; 2 Laboratory of Xinjiang Endemic and Ethic Diseases, Shihezi University, Shihezi, 832000 Xinjiang China; 3 Department of Physiology and Pathophysiology, Tianjin Medical University, Tianjin, 300000 China

**Keywords:** Type 2 diabetes, Obesity

## Abstract

**Objective:**

In our previous study, we identified a notable increase in miR-548ag content after obesity, which contributes to the progression of Type 2 diabetes Mellitus(T2DM) through the up-regulation of Dipeptidyl Peptidase-4(DPP4) expression within the liver. However, the precise molecular mechanisms underlying the upregulation of DPP4 by miR-548ag remain elusive. Mature miRNAs rich in GU sequences can activate the TLR(7/8)/NF-κB signalling pathway, which transcriptionally activates DPP4 expression. Notably, the proportion of GU sequences in hsa-miR-548ag was found to be 47.6%. The study proposes a hypothesis suggesting that miR-548ag could potentially increase DPP4 expression in hepatocytes by activating the TLR(7/8)/NF-κB signalling pathway.

**Methods:**

Male C57BL/6J mice were fed normal chow diet (NCD, *n* = 16) or high-fat diet (HFD, *n* = 16) for 12 weeks. For a duration of 6 weeks, NCD mice received intraperitoneal injections of a miR-548ag mimic, while HFD mice and db/db mice (*n* = 16) were administered intraperitoneal injections of a miR-548ag inhibitor. qRT-PCR and Western Blot were used to detect the expression level of miR-548ag, DPP4 and the activation of TLR(7/8)/NF-κB signalling pathway. HepG2 and L02 cells were transfected with miR-548ag mimic, miR-548ag inhibitor, TLR7/8 interfering fragment, and overexpression of miR-548ag while inhibiting TLR7/8, respectively.

**Results:**

(1) We observed elevated levels of miR-548ag in the serum, adipose tissue, and liver of obese mice, accompanied by an upregulation of TLR7/8, pivotal protein in the NF-κB pathway, and DPP4 expression in the liver. (2) miR-548ag promotes DPP4 expression in hepatocytes via the TLR(7/8)/NF-κB signalling pathway, resulting in a reduction in the glucose consumption capacity of hepatocytes. (3) The administration of a miR-548ag inhibitor enhanced glucose tolerance and insulin sensitivity in db/db mice.

**Conclusions:**

MiR-548ag promotes the expression of DPP4 in hepatocytes by activating the TLR(7/8)/NF-κB signalling pathway. MiR-548ag may be a potential target for the treatment of T2DM.

## Introduction

Type 2 diabetes mellitus (T2DM) is a chronic metabolic disorder characterised by insulin resistance (IR), elevated blood glucose levels, and impaired insulin secretion. According to the International Diabetes Federation (IDF), approximately 537 million people with diabetes were diagnosed in the global adult population (20–79 years old) in 2021 and are expected to reach 783 million in 2045 [1, 2]. Epidemiological evidence indicates that nearly 90% of T2DM patients are associated with overweight or obesity [3, 4], and the higher the body mass index, the higher the prevalence of T2DM [5, 6]. Despite numerous studies highlighting obesity as a critical risk factor for the development of insulin resistance (IR) and T2DM, the precise molecular mechanisms remain incompletely understood.

Dysfunctional adipose tissue significantly contributes to the onset of obesity-associated T2DM, primarily via disrupted adipokine secretion resulting in IR across pivotal metabolic tissues—adipose tissue itself, the liver, and skeletal muscle [7]. Differential expression of micro-ribonucleic acids (miRNAs) has emerged as a crucial factor in the development of T2DM in obese state, exerting influence over diverse signalling pathways [8–11]. Circulating exosome miRNAs are primarily derived from adipose tissue and act as novel adipokines that regulate the function of adjacent or distant tissues [7, 12]. Therefore, identifying new adipose tissue-derived miRNAs and elucidating their specific mechanisms of action in relation to glucose metabolism and IR will provide a new basis for preventing and treating obesity-induced T2DM.

In our other research, we used SNP chip technology found that miR-548ag is positively correlated with obesity signs and biochemical indicators of 1053 individuals. In addition, we have previously documented the pioneering discovery that elevated levels of adipose tissue-derived miR-548ag subsequent to obesity can induce an upregulation in Dipeptidyl Peptidase-4 (DPP4) expression within liver tissues [13]. DPP4, a serine protease primarily expressed in liver tissues, exhibits significant involvement in conditions like obesity, diabetes, and cancer. The DPP4 inhibitor (gliptin) has become an innovative oral therapeutic agent for the treatment of type 2 diabetes mellitus (T2DM) [14–17]. However, recent investigations have revealed significant adverse effects attributed to DPP4 inhibitors. For example, Mu-Chi Chung et al. found that 5.25% of diabetic patients treated with DPP4 inhibitors had gout symptoms [18]. In addition, cumulative use of DPP4 inhibitors significantly increased the incidence of heart failure, myocardial infarction and stroke in diabetic patients [19]. In addition, such cumulative use has been implicated in promoting susceptibility to infectious diseases and significantly increasing the incidence of venous thrombosis in patients [20].

There is increasing evidence to suggest that miRNAs could serve as biomarkers for disease and therapeutic response. Additionally, several miRNA-based therapies have advanced to preclinical and clinical stages [21]. In a previous study, we validated the efficacy of a miR-548ag inhibitor in enhancing insulin sensitivity and inducing hypoglycaemia effects by downregulating DPP4 expression in liver tissues of db/db mice [13]. This finding highlights the potential of miR-548ag as a viable molecular target for the treatment of T2DM. Therefore, it is crucial to clarify the exact molecular mechanisms that enable miR-548ag-induced upregulation of DPP4 expression. Our previous study indicated that DPP4 upregulation by miR-548ag may be due to DNMT3B inhibition [13], but other regulatory mechanisms require further investigation. This exploration promises to provide insights into the biological role of miR-548ag in vivo and its relevance for clinical implementation of miR-548ag inhibitors. This could pave the way for potential application in treating T2DM.

In contrast to their traditional role in post-transcriptional gene regulation, miRNAs possess the capacity to function as direct physiological ligands for specific RNA receptors. Notably, miR-21-5P (31.8% GU), miR-29a-5P (36% GU), and miR-122 (63.6% GU) have been shown to interact with TLR7/8, thereby activating downstream signalling components [22]. With a GU sequence percentage of 47.6%, hsa-miR-548ag (AAAGGUAAUUUGUGGUUUCUGC) has the potential to function as a physiological ligand for TLR7/8. TLR7/8 is the primary Toll-like receptor that recruits myeloid differentiation primary response gene 88 (MyD88), which activates nuclear factor-κB (NF-κB) and subsequently promotes downstream release of inflammatory cytokines [23]. The promoter region of the DPP4 gene contains two NF-κB binding sites, which are downstream targets of NF-κB [24]. These findings suggest that miR-548ag may promote DPP4 expression by affecting the TLR(7/8)/NF-κB signalling pathway.

In our current study, we found that miR-548ag levels were elevated in the serum, adipose tissue, and liver of obese mice. Additionally, we observed an upregulation of TLR7/8 and DPP4 expression in liver tissue, both of which are pivotal components of the NF-κB pathway. Further experimentation in HepG2 and L02 cells provided additional evidence that miR-548ag can upregulate DPP4 expression in hepatocytes by activating the TLR(7/8)/NF-κB pathway. Furthermore, the study revealed that inhibiting miR-548ag through intraperitoneal administration significantly improved glucose tolerance and insulin sensitivity in db/db mice. This was achieved by downregulating hepatic DPP4 expression. These findings suggest that miR-548ag inhibition could be a promising approach for treating type 2 diabetes mellitus (T2DM) by inhibiting DPP4.

## Methods

### Animal care

C57BL/6 male mice were obtained from HUNAN SJA Laboratory Animal Co, LTD. (Hunan, China), and *Lepr*^db/db^, *Lepr*^db/–^male mice were obtained from the Model Animal Research Centre of Changzhou Cavens (Changzhou, China). The mice were housed in groups of 5 animals/cage on a 12 h light/dark cycle in an SPF facility at 22–24 °C and 40–50% humidity. In order to reduce the number of animal sacrifices and ensure the success rate of operation in experimental mice, we adopted a small sample size design. Before group feeding, all male mice were randomly assigned to test and control groups, and both groups were fed ad libitum in order to ensure normal survival of the mice. In male C57BL/6 mice raised on 60% high-fat diet, the body weight and Lee’s index of mice in the high-fat diet group increased significantly (*P* < 0.05) compared with those in the normal diet group; and the weights of liver and various parts of adipose tissues (Epi, Sub, PAT, and MAT), FBG, as well as the serum TG, TC, LDL-C, and FFA content were significantly higher than those in the normal diet group (*P* < 0.05). It was suggested that the diet-induced obese mouse model was successfully constructed.All animal care and handling were carried out according to the international laws and policies, and all animal experiments were approved by the animal ethics committee of the first affiliated Hospital of Shihezi University (A2019–087–01).

### Cell culture

HepG2 and L02 cells were purchased from the Cell Bank of the Chinese Academy of Sciences (Shanghai, China), and were cultured in 25 mmol/L glucose Dulbecco’s modified Eagle’s medium (DMEM, Gbico) supplemented with 10% foetal bovine serum and 1% penicillin/streptomycin (100 µg/mL) at 37 °C in a humidified atmosphere with 5% CO2. After transfection, RNA and protein were extracted or insulin sensitivity assay was performed. 293A cells were purchased from Procell. (Wuhan, China), and were cultured in 25 mmol/L glucose Dulbecco’s modified Eagle’s medium (DMEM, Gbico) supplemented with 10% foetal bovine serum and 1% penicillin/streptomycin (100 µg/mL) at 37 °C in a humidified atmosphere with 5% CO2.

### Adenovirus amplification and purification

The miRNA-548ag mimic adenovirus vector and inhibitor used in this study were constructed by Shanghai Genepharma. The adenovirus was amplified in 293A cells. In brief, 293A cells cultured in 10% foetal bovine serum about 90% confluence were added with adenovirus seed. 48 h post-infection, visible regions of cytopathic effect (CPE) are observed. Harvest adenovirus stock until approximately 90% CPE is observed (typically 72 h post-infection). The cell suspension was freeze-thawed repeatedly 3 times in a methanol dry ice mixing bath and 37 °C water bath, and the supernatant was collected by centrifugation, which can be frozen at −80 °C for long-term preservation. For specific purification procedures, we referred to the instructions of ViraTrap “M Adenovirus Purification Maxiprep KitViraTrap” adenovirus mass purification kit, and the reagent dosage was adjusted according to the proportion of virus suspension quantity.

### Intraperitoneal injection of adenovirus vector in mice

Sixteen 4-week-old C57BL/6 male mice were fed with normal diet (D12450J, 10% energy from fat) until the 16th week. They were divided into two groups: intraperitoneal injection of empty adenovirus group (*n* = 8) and intraperitoneal injection of adenovirus particles encoding miR-548ag -mimic group (*n* = 8, 1 × 10^11^ VP/ mice, once a week) for 6 weeks. Sixteen 4-week-old C57BL/6 male mice were fed with high-fat diet (D12494, 60% energy from fat) until the 16th week. They were divided into two groups: intraperitoneal injection of empty adenovirus group (*n* = 8) and intraperitoneal injection of adenovirus particles with miR-548ag-inhibitor group (*n* = 8, 1 × 10^11^ VP/ mice, once a week) for 6 weeks. Twelve 4-week-old male db/db mice were reared, acclimatised and fed for 1 week and then divided into an intraperitoneally injected empty adenovirus group (*n* = 6) and an intraperitoneally injected miR-548ag inhibitor adenoviral vector group (*n* = 6, 1 × 1011 VP/pupil, 1 injection/week) for 6 consecutive weeks.

### Cell transfection

Lipofectamine 2000 (Catalogue#: 11668–019; Invitrogen, USA) was used to transfect miR-548ag-mimic, miR-548ag-inhibitor and TLR7/8 interference fragment. HepG2 cells were transfected with miR-548ag-mimic, miR-548ag-inhibitor, TLR7/8 interference fragment at concentrations of 50 nM, 75 nM and 80 nM respectively. L02 cells were transfected with miR-548ag-mimic, miR-548ag-inhibitor, TLR7/8 interference fragment were at concentrations of 50 nM, 75 nM and 80 nM respectively. After 4–6 h of transfection, the transfection reagent was replaced with DMEM containing 10% foetal bovine serum. 24 h later, RNA or protein was extracted,or other treatments were performed.

### Cellular glucose consumption and insulin sensitivity assay

Cellular glucose consumption assay: After 12 h of serum-free starvation, cells were transfected with mimic/inhibitor for 24 h, and the culture medium was collected for measuring glucose concentration using the glucose oxidase method.

Cellular insulin sensitivity assay: After 12 h of serum-free starvation, cells were transfected with mimic/inhibitor for 24 h, and Cells were continuously stimulated with 100 nmol/L insulin + low-glucose DMEM (1 g/L) + 10% FBS mixed medium. Culture medium was collected at 0 min, 15 min, 30 min, 45 min, 60 min, 90 min, and 120 min, and glucose concentration was measured.

### Western blotting and antibodies

Total proteins from the liver and HepG2, L02 cells were extracted in RIPA Lysis Buffer (Cat#R0010, Solarbio) containing 1% PMSF (Cat#P8340, Solarbio). Lysates were then quantitated and equal amounts of protein were subjected to SDS-PAGE and immunoblotted with antibodies against GAPDH, p65, p-p65, IκB, p-IκB and DPP4. Antibodies against GAPDH (Cat#4967s) were from ZSGB-BIO, antibodies against DPP4 (ab23841) were from Abcam. And antibodies against p65(4764), p-p65 (3033), IκB (4812) and p-IκB (2859) were from Cell Signalling.

### Real-time PCR

The miRcute Plus microRNA first-strand cDNA kit and miRcute plus microRNA qPCR Kit used in the experiment were purchased from Beijing TIANGEN, the total mRNA reverse transcription kit was purchased from American Thermo Fisher Scientific, and the real-time quantitative PCR kit was purchased from German QIAGEN. The operation was performed according to the instructions, the primers are shown in Supplementary Table 1.

### Plasmid amplification and extraction

The bacteria were incubated in LB medium (peptone: 10 g/L, yeast powder: 5 g/L, NaCl: 10 g/L). and after adding antibiotics, the bacteria were enriched in a constant temperature shaker for 12 hours. The plasmid was extracted according to the instructions of the plasmid extraction kit (TIANGEN).

### Intraperitoneal glucose-tolerance and insulin sensitivity tests in mice

Glucose-tolerance tests were performed in mice after 12-h overnight fasting. After determination of FBG levels, an intraperitoneal bolus of 2 g glucose per kg body weight was administered to each mouse. Blood glucose levels were detected after 15, 30, 45, 60, and 120 min.

Insulin-sensitivity tests were performed in mice after 6-h fasting. After determination of FBG levels, an intraperitoneal bolus of 0.5 UI insulin per kg body weight was administered to each mouse. Blood glucose levels were detected after 15, 30, 45, 60, and 120 min.

### Detection of DPP4 in human serum

DPP4 in the serum of normal subjects(*n* = 20) and subjects with obesity (*n* = 20) was detected. (According to China’s BMI classification: Low weight: BMI < 18.5 kg/m^2^; Normal weight: 18.5 kg/m^2^ ≤ BMI < 24 kg/m^2^; Overweight: 24 kg/m^2^ ≤ BMI < 28 kg/m^2^; Obese: BMI ≥ 28 kg/m^2^). Human DPP4/CD26 ELISA kit was purchased from proteintech. Add 100 µL of sample diluent to the zero well and 100 µL of gradient diluted standard or sample to be tested to the remaining wells, incubate at 37 °C for 2 h, then wash the plate. After shaking off the liquid and tapping the slats, add 100 µL of HRP-labelled detection antibody (1×) to each well and incubate for 40 min at 37 °C. Add 100 µL of TMB Developing Solution to each well and develop the colour for 15–20 min at 37 °C, protected from light, and add 100 µL of Final Solution to each well to change the colour from blue to grey. Measure the optical density (OD) of each well at 450 nm with an enzyme counter using 630 nm as the calibration wavelength.

### DPP4 inhibitor and miR-548ag inhibitor administration

Eighteen 4-week-old male C57BL/6 mice, fed a high-fat diet until week 8, were divided into a blank control group (*n* = 6), a tail-vein injection of miR-548ag inhibitor adeno-associated virus group (*n* = 6, 1 × 10^12^vg/ml) and a gavage of DPP4 inhibitor group (*n* = 6, 30 mg/kg for 4 weeks, 3 times per week gavages). The consent form and ethical approval were provided by the Medical Ethics Committee at the First Affiliated Hospital, Shihezi University School of Medicine (reference number 2019–029–01).

### Blinding

Four researchers were involved in this animal study. The first researcher was responsible for group feeding and numbering of the mice, the second researcher was responsible for injecting the relevant agents (grouping of mice unknown), the third researcher was responsible for taking gross photographs of the mice and surgical dissection of the tissues (grouping of mice unknown), and the fourth researcher was responsible for analysing the levels of expression of the factors in the tissues according to the numbering (grouping of mice unknown), and sending the data to the first researcher for Analysis.

### Statistical analyses

The SPSS statistical package (version 17.0, SPSS Inc, Chicago, IL, USA) was used for statistical data analyses. For data that fitted a normal distribution, statistical differences between groups were determined using an unpaired Student’s *t* test. For data not fitting a normal distribution, a rank-sum test was performed. Chi square test was used for comparison of counting data. *P* < 0.05 was considered statistically significant.

## Results

### The expression of miR-548ag, TLR7/8, key proteins of the NF-κB pathway, and DPP4 in liver tissues markedly increased after obesity

**Fig. 1:**
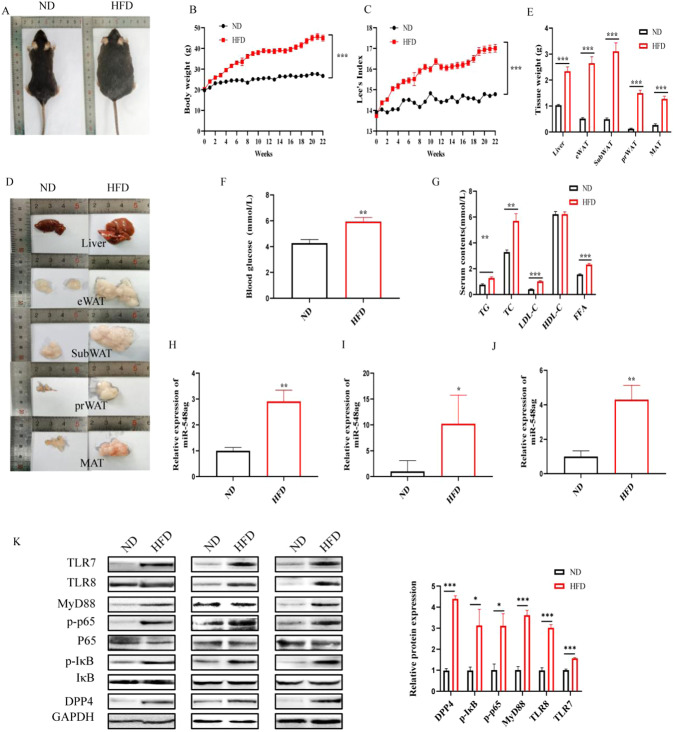
The expression levels of miR-548ag, TLR7/8 and key proteins of NF-κB pathway and DPP4 in liver tissues of obese mice were detected. **A** General morphology of mice; **B** Body weight of mice; **C** Lee’s index of mice; **D** Weight of mice liver and adipose tissue; **E** Morphology of mice liver and adipose tissue; **F** Fasting blood glucose; **G** Serum lipids content; **H**–**J** Content of miR-548ag in epididymal white adipose tissue, serum and liver tissue of mice; **K** Protein expression level of mice in liver tissue. (t-test, *P < 0.05, **P < 0.01, ***P < 0.001).

After 12 weeks on a high-fat diet(HFD) containing 60% fat, mice in the HFD group showed a significant increase in body weight and Lee’s index. In addition, the weights of liver, mesenteric white adipose tissue (MesWAT), perirenal white adipose tissue (PerWAT), epididymal white adipose tissue (EpiWAT) and subcutaneous white adipose tissue (SubWAT) were significantly higher compared to the normal diet(ND) group (Fig. 1A–E). Moreover, blood glucose, FFA, TG, TC, HDL, and LDL levels were markedly elevated in the HFD group (Fig. 1F, G). Notably, the levels of miR-548ag in EpiWAT, serum, and liver tissues of mice in the HFD group exhibited a significant increase, while the protein expression levels of TLR7/8, a key protein of the NF-κB pathway, and DPP4 in liver tissues were also significantly elevated (Fig. 1H–K). All these differences were statistically significant (*P* < 0.05).

### Overexpression of miR-548ag up-regulates the expression of TLR7/8, key proteins of the NF-κB pathway, and DPP4 in liver tissues, leading to decreased glucose tolerance and insulin sensitivity in NCD mice

**Fig. 2:**
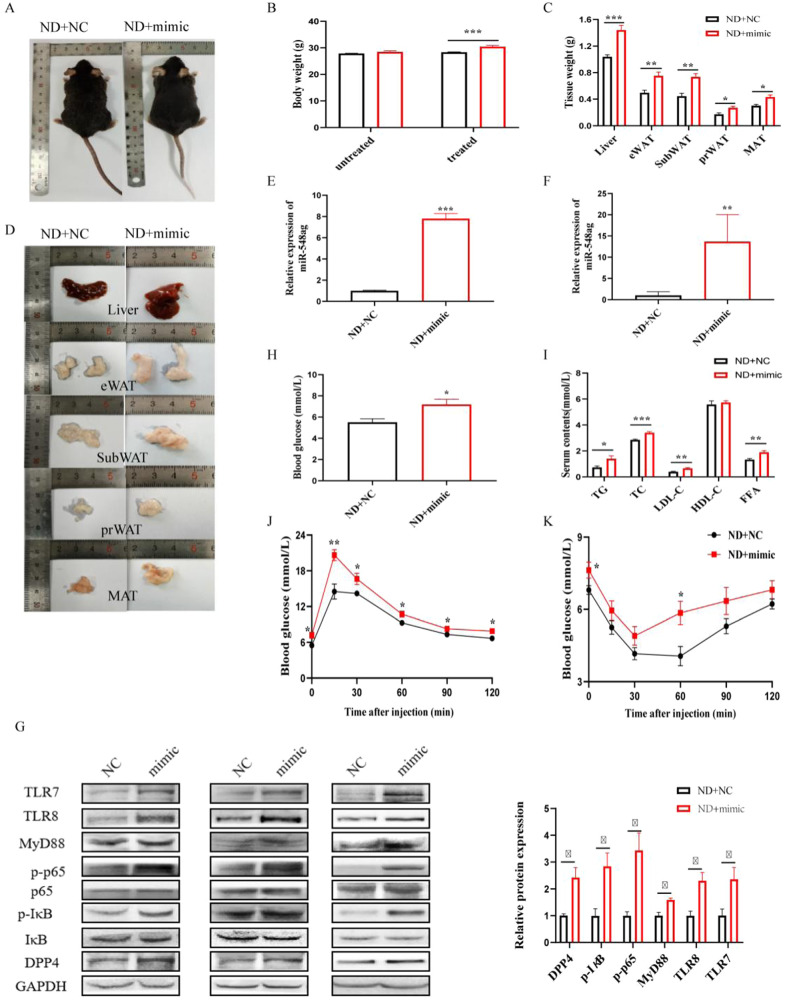
miR-548ag upregulates the expression of TLR7/8, NF-κB pathway key proteins and DPP4 in liver tissues and inhibits glucose tolerance and insulin sensitivity in mice. **A** General view of mice; **B** Body weight of mice; **C** Weight of mice liver and adipose tissue; **D** General morphology of mice liver and adipose tissue; **E**, **F** Expression level of miR-548ag in mice serum and liver tissues; **G** Protein expression level in mice liver tissue; **H** Fasting blood glucose of mice; **I** Serum lipids content of mice; **J** Glucose tolerance of mice; **K** Insulin sensitivity of mice. (Rank sum test and t-test, *P < 0.05, **P < 0.01, ***P < 0.001, difference was statistically significant).

After 6 weeks of intraperitoneal injection of an adenoviral vector overexpressing miR-548ag in mice from the ND group, significant changes were observed. Compared to the control group, the mice exhibited significantly higher body weights, as well as increased weights of liver and adipose tissue (Fig. 2A–D). There was also a substantial increase in miR-548ag expression in serum and liver tissues (Fig. 2E, F), along with significantly higher protein expression levels of TLR7/8, key proteins of the NF-κB pathway, and DPP4 in liver tissues (Fig. 2G). Importantly, these mice displayed elevated blood glucose levels, reduced glucose tolerance, impaired insulin sensitivity, and increased lipid levels such as TC, TG, FFA, and LDL (Fig. 2H–K). All these differences were statistically significant (*P* < 0.05).

### miR-548ag inhibitor down-regulates the expression of TLR7/8, key proteins of the NF-κB pathway, and DPP4 in liver tissues, improving glucose tolerance and insulin sensitivity in obese and db/db mice

**Fig. 3:**
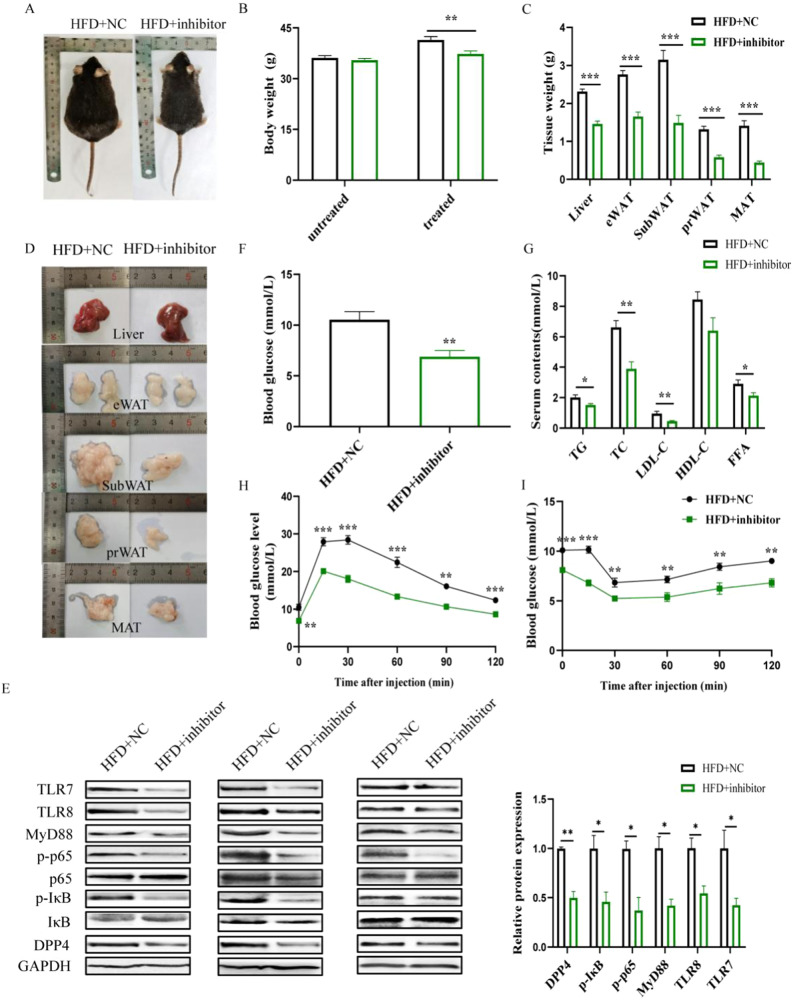
miR-548ag inhibitor down-regulates TLR7/8, NF-κB pathway key proteins and DPP4 expression in liver tissues of obese mice. **A** General view of mice; **B** Body weight of mice; **C** Weight of mice liver and adipose tissue; **D** General morphology of mice liver and adipose tissue; **E** Protein expression level in mice liver tissue. **F** Fasting blood glucose of mice; **G** Serum lipids content of mice; **H** Glucose tolerance of mice; **I** Insulin sensitivity of mice. (t-test, **P < 0.01, ***P < 0.001).

Following a 6-week administration of intraperitoneal injections of miR-548ag inhibitor adenoviral vectors to mice in the HFD group, notable changes were observed compared to the control group. The mice exhibited a significant reduction in body weights, as well as decreased weights of liver and adipose tissue (Fig. 3A–D). Moreover, there was a significant decrease in the protein expression levels of TLR7/8, key proteins of the NF-κB pathway, and DPP4 in the liver tissues (Fig. 3E). Additionally, the mice showed a significant reduction in blood glucose levels, improved glucose tolerance, enhanced insulin sensitivity, and decreased lipid levels such as TC, TG, and LDL (Fig. 3F–I). All these differences were statistically significant (*P* < 0.05).

**Fig. 4:**
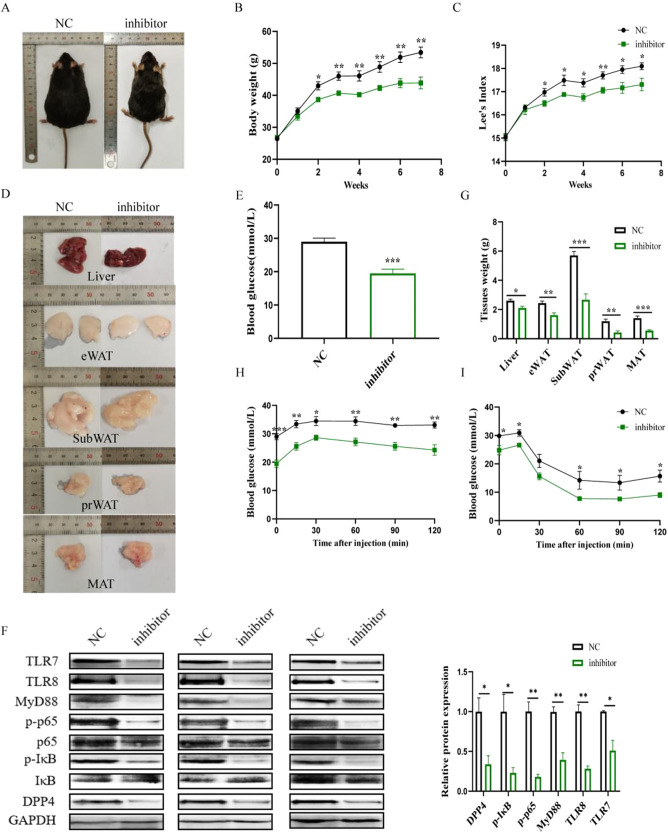
miR-548ag inhibitor down-regulates TLR7/8, NF-κB pathway key proteins and DPP4 expression in liver tissues of db/db mice. **A** General view of mice; **B** Body weight of mice; **C** Weight of mice liver and adipose tissue; **D** General morphology of mice liver and adipose tissue; **E** Weights of liver and adipose tissueSerum lipids content of mice. **F** Protein expression level in mice liver tissue; **G** Fasting blood glucose of mice; **H** Glucose tolerance of mice; **I** Insulin sensitivity of mice. (*t*-test, **P < 0.01, ***P < 0.001).

Similarly, in db/db mice, the administration of intraperitoneal injections of miR-548ag inhibitor adenoviral vector for 6 weeks resulted in significant reductions in weight, Lee’s index, as well as liver and adipose tissue weights compared to the control group (Fig. 4A–E). Furthermore, the protein expression levels of TLR7/8, key proteins of the NF-κB pathway, and DPP4 were significantly reduced in the liver tissues (Fig. 4F). Notably, the intraperitoneal injection of miR-548ag inhibitor significantly reduced blood glucose and lipid levels in db/db mice, leading to a significant improvement in glucose tolerance and insulin sensitivity (Fig. 4G–I). All these differences were statistically significant (*P* < 0.05).

### miR-548ag promotes DPP4 expression in hepatocytes through activation of TLR(7/8)/NF-κB pathway

**Fig. 5:**
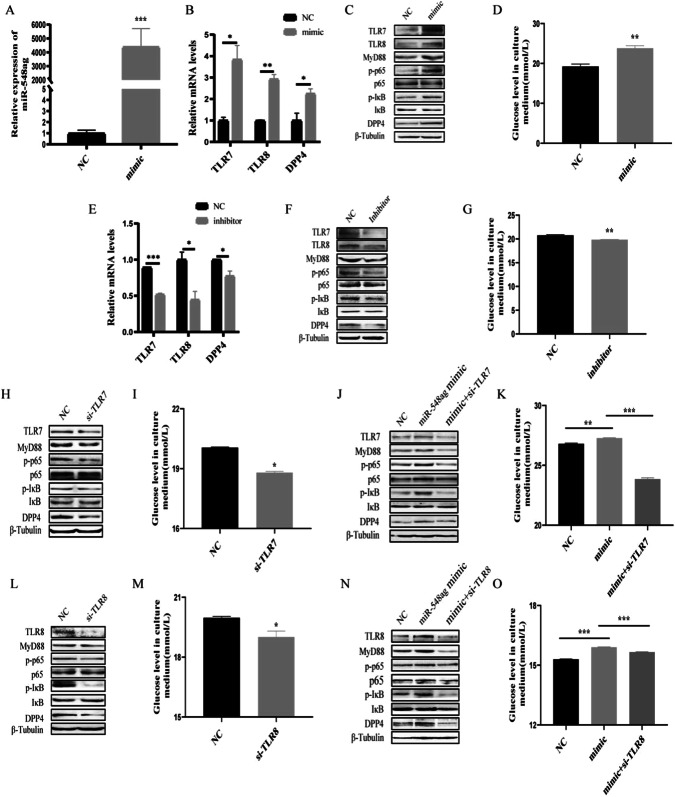
miR-548ag promotes DPP4 expression in HepG2 through activation of TLR(7/8)/NF-κB pathway. mRNA expression levels of (**A**) miR-548ag, (**B**) TLR7/8, DPP4 in HepG2 cells after transfection with miR-548ag; **C** Protein expression levels of TLR(7/8), key proteins of NF-κB pathway, DPP4 in HepG2 cells after transfection with miR-548ag (**D**) HepG2 glucose consumption levels. **E** mRNA expression levels of TLR7/8, DPP4 in HepG2 cells after transfection with miR-548ag inhibitor; **F** Protein expression levels of TLR(7/8), key proteins of NF-κB pathway, DPP4 in HepG2 cells after transfection with miR-548ag inhibitor; **G** HepG2 glucose consumption levels; **H** Protein expression levels of TLR(7/8), key proteins of NF-κB pathway, DPP4 in HepG2 cells after transfection with TLR7 interference fragment; **I** Cell glucose consumption of HepG2; **J** The protein expression levels of HepG2 cells were transfected with TLR7 interference fragments after miR-548ag was modulated; **K** Glucose consumption of HepG2; **L** Protein expression level of HepG2 cells after transfection with TLR8 interference fragment; **M** Cell glucose consumption of HepG2; **N** Protein expression levels of HepG2 cells after transfection of TLR8 interference fragments with up-regulated miR-548ag; **O** Glucose consumption of HepG2 (Rank sum test, *P < 0.05, **P < 0.01, ***P < 0.001, the difference was statistically significant).

**Fig. 6:**
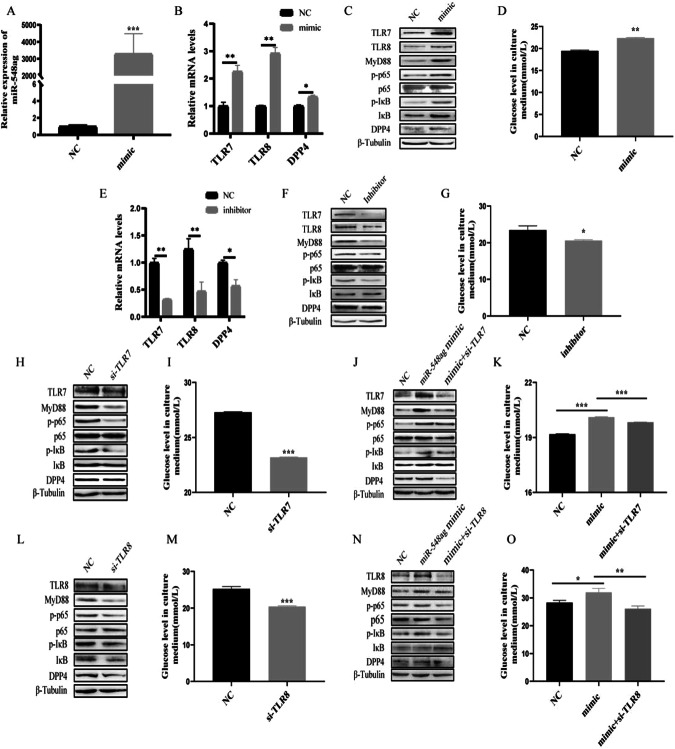
miR-548ag promotes DPP4 expression in L02 through activation of TLR(7/8)/NF-κB pathway. mRNA expression levels of (**A**) miR-548ag, (**B**) TLR7/8, DPP4 in L02 cells after transfection with miR-548ag; **C** Protein expression levels of TLR(7/8), key proteins of NF-κB pathway, DPP4 in L02 cells after transfection with miR-548ag (**D**) L02 glucose consumption levels; **E** mRNA expression levels of TLR7/8, DPP4 in L02 cells after transfection with miR-548ag inhibitor; **F** Protein expression levels of TLR(7/8), key proteins of NF-κB pathway, DPP4 in L02 cells after transfection with miR-548ag inhibitor; **G** L02 glucose consumption levels; **H** Protein expression levels of TLR(7/8), key proteins of NF-κB pathway, DPP4 in L02 cells after transfection with TLR7 interference fragment; **I** Cell glucose consumption of L02; **J** The protein expression levels of L02 cells were transfected with TLR7 interference fragments after miR-548ag was modulated; **K** Glucose consumption of L02; **L** Protein expression level of L02 cells after transfection with TLR8 interference fragment; **M** Cell glucose consumption of L02; **N** Protein expression levels of L02 cells after transfection of TLR8 interference fragments with up-regulated miR-548ag; **O** Glucose consumption of L02 (Rank sum test, *P < 0.05, **P < 0.01, ***P < 0.001, the difference was statistically significant).

HepG2 and L02 cells were transfected with 50 nM miR-548ag mimic (Supplementary Fig. S1), resulting in successful overexpression of miR-548ag (Figs. 5A and 6A). There was a significant increase in the mRNA and protein expression levels of TLR7/8, key proteins of the NF-κB pathway, and DPP4 in the cells (Figs. 5B, C and 6B, C), accompanied by a significant inhibition of the cells’ glucose-consuming ability (Figs. 5D and 6D). All these differences were statistically significant (*P* < 0.05). Moreover, transfection of 75 nM miR-548ag inhibitor into HepG2 and L02 cells resulted in a notable reduction in the mRNA and protein expression levels of TLR7/8, key proteins of the NF-κB pathway, and DPP4 (Figs. 5E, F and 6E, F). Additionally, it significantly promoted the glucose-consuming ability of the cells (Figs. 5G and 6G). All these differences were statistically significant (*P* < 0.05).

Compared to the control group, the overexpression of miR-548ag significantly increased the protein expression levels of TLR7/8, p65, p-p65, IκB, p-IκB, MyD88 and DPP4. Furthermore, the effects of miR-548ag on the TLR7/8 and NF-κB pathway were significantly reversed by interfering with TLR7/8. Moreover, interference with TLR7/8 significantly improved the detrimental effects of miR-548ag overexpression on cellular glucose-consuming ability (Figs. 5H–O, 6H–O). All these differences were statistically significant (*P* < 0.05).

### The levels of miR-548ag and DPP4 were significantly positively correlated in human serum

**Fig. 7:**
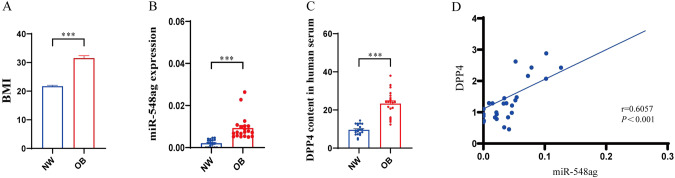
The levels of miR-548ag and DPP4 were significantly positively correlated in human serum. **A** BMI in normal weight individuals and individuals with obesity; **B** Serum miR-548ag levels were significantly higher in individuals with obesity (*n* = 20) than in normal-weight individuals; **C** Serum DPP4 levels were significantly higher in individuals with obesity (*n* = 20) than in normal-weight individuals; **D** There was a positive correlation between serum miR-548ag and DPP4 levels in individuals with obesity (*n* = 40). (*t*-test, *P < 0.05, **P < 0.01, ***P < 0.001; Pearson’s correlation analysis).

miR-548ag and DPP4 in human serum were detected. Serum miR-548ag and DPP4 levels were significantly higher in obese individuals (*n* = 20) than in normal weight individuals (Fig. 7B, C). Furthermore, there was a positive correlation between serum miR-548ag and DPP4 levels in individuals with obesity(Fig. 7D). All these differences were statistically significant (*P* < 0.001).

### Comparison of the hypoglycaemic effects of DPP4 inhibitor and miR-548ag inhibitor

**Fig. 8:**
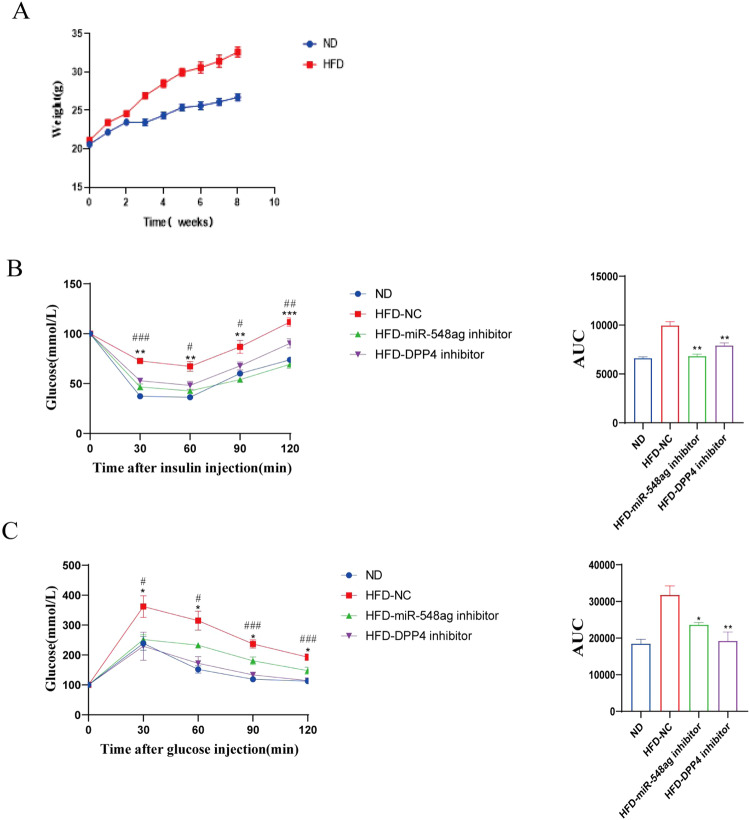
Injection of miR-548ag inhibitor and gavage of DPP4 inhibitor were both effective in improving glucose tolerance and insulin sensitivity in mice. **A** Body weight of mice; **B** ITT and area under the ITT curve for injection of miR-548ag inhibitor and gavage of DPP4 inhibitor; **C** GTT and area under the GTT curve for injection of miR-548ag inhibitor and gavage of DPP4 inhibitor. (*t*-test, *P < 0.05, **P < 0.01, ***P < 0.001).

Eighteen 4-week-old male C57BL/6 mice, fed a high-fat diet until week 8, were divided into a blank control group (*n* = 6), a tail-vein injection of miR-548ag inhibitor adeno-associated virus group (*n* = 6, 1 × 10^12^vg/ml) and a gavage of DPP4 inhibitor group (*n* = 6, 30 mg/kg for 4 weeks, 3 times per week gavages). After comparing the ITT and GTT of mice in different treatment groups, it was found that glucose tolerance and insulin sensitivity were significantly improved in mice given DPP4 inhibitor by gavage and miR-548ag inhibitor by tail vein injection compared with control obese mice. Both interventions were equally effective at improving glucose tolerance and insulin sensitivity in mice (Fig. 8A–C). All these differences were statistically significant (*P* < 0.001).

## Discussion

Several studies have demonstrated that obesity alters the endocrine function of adipose tissue. Novel adipokine miRNAs are produced, which induce insulin resistance (IR) by inhibiting the expression of target genes, ultimately leading to type 2 diabetes mellitus (T2DM) [12, 25–29]. Searching for key differentially expressed miRNAs during the development of obesity and T2DM and elucidating their possible mechanisms leading to dysfunctions in the body’s glucose and lipid metabolism will provide a theoretical basis for the prevention and treatment of obesity-induced T2DM.

In a previous study, we discovered a significant positive correlation between miR-548ag and fasting blood glucose (FPG) levels in 1053 subjects’ genomic DNA using single nucleotide polymorphism (SNP) microarray chips. Subsequent research revealed that increased expression of miR-548ag can disrupt glucose metabolism by up-regulating the expression of DPP4, which contradicts the classical pathway of miRNA function. Our previous study aimed to clarify the molecular mechanism by which miR-548ag up-regulates DPP4 expression. The promoter region of the *DPP4* gene had a CG content greater than 40%, indicating that methylation may regulate DPP4 expression. Bioinformatic prediction, combined with dual-luciferase reporter gene experiments, confirmed that miR-548ag could target and inhibit the expression of DNMT3B. However, subsequent studies have shown that miR-548ag does not upregulate DPP4 expression by affecting the methylation status of the promoter region of the DPP4 gene [13]. These results suggest that there may be other molecular regulatory mechanisms responsible for the upregulation of DPP4 expression by miR-548ag.

The traditional role of miRNA is to reduce the expression level of specific target genes by binding to the 3’UTR region of downstream target genes, i.e., it is a negative regulatory relationship with downstream target genes [30–32]. However, miR-548ag affects glucose metabolism by upregulating DPP4 expression, and its specific molecular mechanism needs to be further explored. Currently available studies suggest a total of seven molecular mechanisms of non-classical regulation of miRNAs: (1) pri-miRNAs can be translated into polypeptides; (2) miRNAs can bind to other functional proteins; (3) miRNAs can directly activate TLR-like receptor proteins (4) miRNAs can increase the level of protein expression; (5) miRNAs target the regulation of mitochondrial-associated gene mRNAs; (6) miRNAs can directly activate gene transcription processes; (7) miRNAs can target precursor RNAs that negatively regulate other non-coding RNAs [33–39].

Recent studies have shown that mature miRNAs enriched in GU sequences can also act as physiological ligands for TLR7 and TLR8 to promote immune responses [12]. And Fichtlscherer S et al. found that the promoter region of the DPP4 gene contains two NF-κB binding sites, which have been shown to be the downstream target genes of NF-κB signalling pathway [26]. Hsa-miR-548ag (AAAGGUAAUUGUGGUUUCUGC) has a GU sequence percentage of 47.6%, could also act as the physiological ligand of TLR7/8 to activate the NF-κB signalling pathway to promote DPP4 expression in hepatocytes. It would be a very interesting scientific question. In present study, we verified that miR-548ag can up-regualte DPP4 expression by activating TLR(7/8)/MyD88/NF-κB pathway, and significantly inhibit glucose tolerance and insulin sensitivity of cells and mice in vivo and in vitro. The above results suggest that miR-548ag may also act as an endogenous ligand to up-regulate the expression of DPP4 in liver tissues by activating the TLR7/8-NF-κB signalling pathway. The increase of MyD88 expression level and the phosphorylation level of NF-κB active subunit p65 is a pivotal marker of TLR7/8 ativation, in present study, we confirmed that miR-548ag can activate TLR7/8 by detecting the above factors. In addition, our study also found that miR-548ag can up-regulate the expression of TLR7/8, the specific molecular mechanism of which needs to be further explored in the future.

Currently, DPP4 inhibitors have been widely used in the treatment of T2DM. However, recent studies have found that DPP4 inhibitors have major side effects, such as gout symptoms, infectious diseases, venous thrombosis, heart failure, myocardial infarction, and stroke in diabetic patients [21–23]. A growing number of studies have shown that miRNAs are potential biomarkers of disease and therapeutic response, and a considerable number of therapies for miRNAs have entered preclinical studies, some of which are in clinical development [24]. In order to preliminarily explore whether miR-548ag inhibitor has a role in improving T2DM phenotype, in this study, after intraperitoneal injection of miR-548ag inhibitor adenoviral vector for 6 weeks, glucose tolerance and insulin sensitivity of the db/db mice were significantly improved, and a single tail vein injection of the miR-548ag inhibitor was as effective as 12 gavages of the DPP4 inhibitor, which suggest that miR-548ag inhibitor may become a novel DPP4 inhibitor and a novel therapeutic agent against T2DM. Further evaluation of the effects of miR-548ag inhibitor in improving the body’s glucose tolerance and insulin sensitivity will provide a theoretical basis for miR-548ag to become a candidate drug target for the clinical treatment of obesity and related metabolic diseases.

In addition, this study confirmed that overexpression of miR-548ag can activate the TLR7/8-NF-κB inflammatory pathway through in vitro and in vivo experiments. We have already shown that excess free fatty acids produced by increased lipolysis after obesity can lead to a chronic low-grade inflammatory state through activation of TLR4/9 [40–42]. The present study demonstrates for the first time that increased circulating miRNAs after obesity can also serve as an important initiator of the induced inflammatory response.

In summary, based on in vitro culture of HepG2 and L02 cells and construction of a diet-induced obese mouse model, the present study demonstrated that miR-548ag can up-regulate the DPP4 expression in liver tissue by activating the TLR(7/8)/NF-κB pathway, which ultimately led to abnormal glucose tolerance and reduced insulin sensitivity. Finally, the therapeutic effects of miR-548ag inhibitor were explored in db/db mice. Based on this study, a thorough evaluation of the diagnostic and therapeutic potential of miR-548ag in obesity and related metabolic diseases could offer novel treatment options and targets.

### Supplementary information

Supplementary Table 1

## Data Availability

The data used in this study are available from the corresponding author on reasonable request.
