# Supplementary material for: miR-548ag promotes DPP4 expression in hepatocytes through activation of TLR(7/8)/NF-κB pathway
Source: Int J Obes (Lond). 2024 Feb 29;48(7):941–53. doi: 10.1038/s41366-024-01504-8 (PMC11217002; doi:10.1038/s41366-024-01504-8)
Supplement: Supplementary file 1 — Supplementary Table 1 [file 41366_2024_1504_MOESM1_ESM.pdf]

Supplementary Table 1

The Primer Sequences of Target Genes for qRT-PCR

Species	Primer	Sequence 5’→3’
Human	Human-*microRNA-548ag*-F	5'- AAAGGUAAUUGUGGUUUCUGC-3'
	Human-*TLR7*-F	5'- CTTTGGACCTCAGCCACAACCA-3'
	Human-*TLR7*-R	5'- CGCAACTGGAAGGCATCTTGTAG-3'
	Human-*TLR8*-F	5'-ACTCCAGCAGTTTCCTCGTCTC-3'
	Human-*TLR8*-R	5'-AAAGCCAGAGGGTAGGTGGGAA-3'
	Human-*DPP4*-F	5'- GGGTCACATGGTCACCAGTG-3'
	Human-*DPP4*-R	5'- TCTGTGTCGTTAAATTGGGCATA-3'
	Human-*GAPDH*-F	5'- TGTGGGCATCAATGGATTTGG-3'
	Human-*GAPDH*-R	5'- ACACCATGTATTCCGGGTCAAT-3'
Mouse	Mus-*DPP4*-F	5'-CACCTCTGATGGAAGCAGCTTC-3'
	Mus-*DPP4*-R	5'-GATAATCGCTGGTCAGAGCTTCG-3'
Micro-RNA-548ag mimic/inhibitor sequence	miR-548ag (mimic)	5'-AAAGGUAAUUGUGGUUUCUGU-3'<br>5'-AGAAACCACAAUUACCUUUUU-3'
	miR-548ag (inhibitor)	5'-GCAGAAACCACAAUUACCUUU-3'
